# Acupuncture with manual and low frequency electrical stimulation as experienced by women with polycystic ovary syndrome: a qualitative study

**DOI:** 10.1186/1472-6882-12-32

**Published:** 2012-04-03

**Authors:** Annika Billhult, Elisabet Stener-Victorin

**Affiliations:** 1 Research and Development Unit of the County Södra Älvsborg, Sven Eriksonsplatsen 4, 503 38 Borås, Sweden; 2 Institute of Neuroscience and Physiology, Department of Physiology, Sahlgrenska Academy, University of Gothenburg, 405 30 Gothenburg, Sweden; 3 Department of Obstetrics and Gynecology, First Affiliated Hospital, Heilongjiang University of Chinese Medicine, Harbin, China

## Abstract

**Background:**

Polycystic ovary syndrome (PCOS) affects 5-10 percent of all fertile women and is associated with anovulation/oligoovulation, hyperandrogenism, and polycystic ovaries. Pharmacological treatment is often effective but associated with unwanted side effects. Acupuncture treatments have been shown to improve menstrual bleeding patterns and ovulation as well as hyperandrogenism, without side effects. The purpose of the present study was to describe the experience of acupuncture for women diagnosed with PCOS.

**Methods:**

Eight women with PCOS living in western Sweden, were interviewed following repeated acupuncture treatments. Data was analyzed using systematic text condensation as described by Malterud.

**Results:**

The experience of acupuncture for women diagnosed with PCOS can be described in five categories; the experience of hope, getting results, feelings of responsibility, skepticism and proof of effect, and feeling normal.

**Conclusion:**

Since acupuncture is a promising treatment for the symptoms of the common syndrome PCOS, the present study adds to the knowledge base by providing the important experiences of patients receiving the treatment. Acupuncture provides a possibility for patients to gain hope as the treatment shows results. The results show that acupuncture empowers the patients to take responsibility for their future well-being, although they may have been initially skeptical to the treatment. Because the syndrome had affected them for some time, even small changes offered a chance for them to feel that their bodies were capable of normal function.

**Trial Registration:**

The trial is registered at Clinical Trials.gov with Identifier number NCT00484705.

## Background

Polycystic ovary syndrome (PCOS) affects 5-10 percent of all women of reproductive age and is associated with anovulation/oligoovulation, hyperandrogenism, and polycystic ovaries (PCO) [1,2]. PCOS is associated with metabolic disturbances including obesity and insulin resistance with a high risk of developing type 2 diabetes, and cardiovascular disease [2]. In addition, women with PCOS display reduced health-related quality of life as well as symptoms of anxiety and depression [3,4]. Despite its high prevalence, there is no gold standard for long term treatment of women with PCOS. Pharmacological treatment is often effective but associated with unwanted side effects. The use of acupuncture among women visiting reproductive endocrinologist/fertility clinics is common and varies from 22% in the United States, to 12.5% in Australia and to 8% in the United Kingdom [5-7]. Repeated acupuncture treatments with a combination of electrical and manual stimulation of the needles has, in both uncontrolled [8,9] and randomized controlled trials been shown to improve menstrual bleeding patterns and ovulation as well as hyperandrogenism [10,11]. Thus acupuncture may be a complement to pharmacological treatment in the relief of PCOS symptoms. However, studying the effect of electro acupuncture on symptoms of PCOS is not enough. A recent qualitative study describes how young women's experiences of living with PCOS are marked by daily physical, social and emotional challenges [12]. No previous study, to our knowledge, has described the experience of receiving electro acupuncture treatment in women diagnosed with PCOS. Thus, the purpose of this study was to describe the experience of acupuncture treatment with a combination of manual and electrical stimulation for patients with PCOS.

## Materials and method

### Participants

Women included in an earlier RCT previously described in detail [10], were recruited until material rich enough for analysis was obtained. Eight women were asked to participate and all eight accepted and were included in the study. All eight women had polycystic ovaries (more than 12 follicles < 9 mm measured with transvaginal ultrasonography), clinical signs of hyperandrogenism (hirsutism: > 8 on Ferriman Gallway score), and oligo-, amenorrhea. They were free from any medications three months entering the study and throughout the study period. They were selected purposively to achieve variation in age. Mean age was 31.9 (23-38) years. Occupations varied including a nurse, nurse's assistant, pharmacist, office clerk, student, bus driver, and engineer. The mean time from last electro acupuncture treatment to time of interview was 6.75 (4-12) months. Women were contacted by letter asking if they were interested in participating in the study. None of the women received acupuncture or any other treatment for PCOS between the time of the intervention and the interview. The study was conducted in accordance with the Declaration of Helsinki and approved by the Ethics Committee, University of Gothenburg, Sweden. All informants signed informed consent before interviews were conducted. The trial is registered at Clinical Trials.gov with Identifier number NCT00484705.

### Treatment

Acupuncture with manual and electrical (low frequency) stimulation of the needles was performed twice weekly for 2 weeks, once weekly for 6 weeks, and once every other week for 8 weeks (total, 14 treatments) by a physical therapist trained in Western medical acupuncture. Each treatment lasted 30 minutes. The protocol was based on our studies in women with PCOS [9,13], experimental studies [14], and clinical experience. The acupuncture points and electrical/manual stimulation applied have previously been described in detail [10,15].

### Data collection

Participants first recieved a letter containing information about the study. They were then contacted by telephone by AB to arrange interviews. They were interviewed after their last treatment using an open interview technique to retrieve data. All participants were invited to elaborate on their experiences by the statement: "Please tell me about your experience of the acupuncture treatment". The interviewer had an open and flexible attitude towards the informant which led the informant to elaborate freely on the phenomena. Follow-up questions such as "How did that feel?" and "Please tell me more" were asked to deepen understanding of the phenomena. Dialogic validation was used throughout the interview to minimize misunderstanding. Interviews took place at the informants' convenience, seven at the university and one at the informants work place. Interviews lasted approximately 30 minutes and were recorded, transcribed verbatim and de-identified by AB.

### Analysis

As the objective of the present study was to describe the experience of acupuncture treatment for women with PCOS, a qualitative research method was chosen. Analysis was carried out using Malterud's systematic text condensation [16], inspired by Giorgi's descriptive phenomenological research method. As the material was vast, only information pertaining to the research question was analysed. The analysis was carried out in four steps. First, interviews were re-read many times for the researcher to become acquainted with the interviews. The first step ended with the researcher moving from a broader perspective of the material to a narrower search for patterns containing information on women's experiences of acupuncture treatment. These patterns were then elaborated on and systemized in step two where meaning units were identified and coded. The process of coding was done using different colors to mark the relevant text. Codes were then decontextualized and joined with others of the same color. Step three meant condensing and summarizing codes. Finally, the codes were generalized and recontextualized. The data now moved from a low level of abstraction to overarching themes ingrained within the concrete facts provided by the data. When analyzing, the researcher adopted a holistic approach as a theoretical framework since the study focused on the complex issues of the human body. The excerpts of the women were originally documented in Swedish and then translated by a native English-speaking person. The study was reported using the RATS guidelines [17].

## Results

When asked to relate their experiences of acupuncture with electrical and manual stimulation of the needles, informants spoke of them in relation to the diagnosis of PCOS. Their descriptions could be summarised in five categories; 1) the experience of hope, 2) acupuncture triggered things, 3) feelings of responsibility, 4) scepticism and proof of effect, and 5) feeling normal.

### The experience of hope

The women were aware of their symptoms as a result of the PCOS diagnosis. They had lived with these problems in varying degrees, and when faced with a possible solution and treatment, developed a sense of hope toward a positive effect. Issues of concern were, for example, the fact that childbearing might never become a reality. One woman expressed this longing for children as:

*"I have always loved children, but when I got this PCOS, I felt that I might never become a mother. This *[the acupuncture] *became some sort of rescue, maybe it will happen despite..." IP 7*

This woman had chosen career before family, but the longing for children had always stayed with her. She was now excited about developing that side of herself as well. The acupuncture treatment gave participants a sense of hope not previously experienced. The general feeling among participants, before entering the study, was that PCOS was untreatable.

### Acupuncture triggered things in my body

Even though not all participants in the study noticed great results, over half of the participants experienced a change in their health status relating to the symptoms caused by PCOS. They associated acupuncture treatment with improvements, for example, in more regular menstrual bleeding, decreased hair growth, improved mood and decreased acne. One woman even got pregnant due to what she believed might be the result of acupuncture treatment. Another woman experienced a decreased craving for sweets:

"*I managed to not eat in the evenings during acupuncture treatment... It felt fantastic not having to provide my body with anything. I had no cravings at all... there was a major difference during the acupuncture treatment, there really was." IP 1*

When beginning the acupuncture treatment, participants had various expectations. As some of them were hopeful for the treatment to have effect on some of their symptoms, hope in relation to decreased hair growth was not expressed. One of the participants who expected results from the treatment to affect, for example, her menstruation cycle expressed the following:

"*My hair growth decreased, and that was... I had never hoped for that..." IP 4*

Willing to be surprised by the effect of the treatment, some of the participants experienced relief in areas not expected.

### A feeling of responsibility came over me as the acupuncture treatment gave me an insight into change

As participants felt that something happened to their bodies, they became inspired to change habits in their lives that could perhaps prolong the positive effects. Acupuncture treatment with manual and electrical stimulation of the needles started a process towards a change that became evident in the results they experienced. Feelings of responsibility to take charge and attempt to influence their condition started with the treatment. The small insight of change they received during treatment was enough to inspire and empower participants.

"*Something happened in my head during treatment. I was afraid when treatment was over because I did not want it *[the cravings] *back; at the same time it was a great opportunity for me to get started" IP 1*

The diagnosis of PCOS could at times even serve as an excuse for the patient's symptoms. To maintain old habits and blame it on PCOS, was common behavior for some until they discovered the powerful effect in the insight of change.

### Skepticism and proof of effect

Some participants in the study had no expectations for the treatment at all. An open attitude was seen where if something changed it did, if not, it did not. Since the basic understanding was that PCOS was something untreatable, they did not expect acupuncture to have any effect. One patient expressed this in the following way:

"I had no expectations whatsoever. Whatever happened happened..." IP5

Scepticism to acupuncture also meant that participants were surprised when, as treatment continued, they experienced change and improvements in the symptoms. Discovering that the body responded convinced them of the possibilities of acupuncture treatment having an effect.

"*I am surprised that the body responded to it *[the acupuncture treatment]*" IP 5*

The proof of effect came as the participants felt a change in their bodies, even though they, in their state of disbelief, could not understand the powerful effect of the needles.

### My body has the possibility of normal functions

An effect of acupuncture treatment such as regaining a regular menstrual period provided relief for the participants. Even though menstruation was experienced as bothersome, it felt good to know the body was working in that sense.

"*It is just a relief to know that it *[the menstruation] *arrives every month. Even if that week is not a fun one...it is good to know that it will be there" IP2*

As opposed to feeling tired, bloated and having a craving for sweets, several of the participants expressed a feeling of wellness strongly connected to bodily functions such as being able to get pregnant, decreased hair growth and smooth skin. Patient self-esteem was also influenced by their bodies being affected by PCOS, especially as visible symptoms, such as acne and excess hair growth, decreased.

## Discussion

This is the first study to describe the experience of receiving acupuncture among women with PCOS. The main findings of the study were the experiences of hope, acupuncture triggered things, feelings of responsibility, skepticism and proof of effect, and feeling normal.

### Study method

As acupuncture with manual and electrical stimulation of the needles by nature involves a therapist, it is possible that the patient-therapist interaction have influenced the results. The treatment was administered by one therapist only, making her personality vital concerning interaction with the informants. This study did not however, investigate patient-therapist interaction.

The time from treatment to interview varied between 4-12 months. It was not possible due to practical circumstances to shorten this gap. It is possible that this may have created some recall bias.

Qualitative method is suitable for investigating the life-world of the informants. As life-world science is complex, it demands the researcher to be stringent and adhere strictly to the study method. It was our intention to describe and follow the method thoroughly in order to increase validity. In order to be open and truly surprised by the informants, bracketing is central to systematic text condensation. To avoid influencing results, the researcher (AB) documented her preconceived thoughts about the phenomenon, and returned to these during the process of the study. In addition, patients diagnosed with PCOS were not included in the clinical experience of AB, and the participants in the present study were, prior to the interviews, unknown to AB.

Since the interview setting was an interpersonal meeting, it is possible that informants expressed only matters that were positive and flattering. However, the informants in this study expressed scepticism and some of them related how they had gained no effect from the treatment at all, implying that their stories were trustworthy.

## Discussion of results

The inclusion criterion for the study was a diagnosis of PCOS. The aim was therefore to illuminate this defined group of women's experiences of receiving 14 acupuncture treatments during a 16-week period. It is the nature of acupuncture to create sensations such as discomfort during needle placement, tension, relaxation and confidence in the therapist. Although the informants in this study also included such descriptions in their stories, the only results presented were those experienced in relation to the fact that the informants were diagnosed with PCOS. In condensing their stories five categories, with no internal order of importance, emerged.

### A feeling of hope

First of all, participants expressed gaining a feeling of hope. They had a preconception that PCOS untreatable. To be included in a study evaluating a promising treatment for PCOS, gave them a sense of it possibly having an effect. Some informants had heard from others that acupuncture may have an effect on PCOS, and some had experience of positive results of acupuncture for other reasons, leading them to hope and expect effects on their symptoms. An earlier study showed that participants included in a placebo-controlled acupuncture study spoke of hope rather than despair [18].

### Acupuncture triggered things

Besides feeling hopeful, acupuncture treatment actually had some effect on the symptoms caused by PCOS. Some women even experienced concrete results such as more regular ovulation and even pregnancy. Acupuncture has been shown to have effect on ovulation and menstrual bleeding patterns in earlier studies [9-11], which confirms the experiences of the participants in this study. Pharmacological treatment in PCOS is symptom oriented and often associated with negative side effects [19,20]. In overweight and obese PCOS women, lifestyle interventions including exercise and diet is the first-line therapy for all PCOS related symptoms [20]. However, for many women this is a very difficult task and complement/alternative is warranted. Importantly, acupuncture is a treatment without unwanted side-effects. Besides effects on menstruation, the women in the present study also experienced effects such as decreased hair growth, decreased cravings for sweets and decreased acne according to the interviews. These results are somewhat surprising since from our quantative measures we could not detect a decrease in their hair growth [10]. However, at the follow-up, 4 months after the last treatment, women receiving acupuncture rated less acne [10] confirming these observations.

### Feeling of responsibility

Acupuncture treatment gave the participants a feeling of responsibility, as they discovered that the treatment had an effect on their bodies. Even if the effect was barely noticeable, it was enough for them to start taking charge of their own well-being. Acupuncture worked as a mechanism providing an energy boost to carry on the positive trend towards feeling better. Evans et al (2011) emphasized the importance of self-care along with the practise of acupuncture, at the same time recognising the difficulties in adhering to such advice [21]. It seems that the acupuncture treatment itself in the present study gave the participants the motivation needed to take responsibility and initiate change. This is in accordance with another study reporting participants taking an active role in life style changes following acupuncture treatment [22]. Colwell et al. (2010) reported, in an uncontrolled trial including participants with PCOS, how they felt empowered by participating in a clinical research study [23]. As the acupuncture treatment of the present study was limited to a specific number of treatments, participants expressed worry that the effect would diminish, providing them with further incentives to prolong the effect by making changes on their own.

### Skepticism and proof of effect

Some participants in this study expressed scepticism to the fact that the acupuncture would actually have any effect on their bodies at all. They had lived with the symptoms of the syndrome for a long time, believing no treatment was available. Allotted to acupuncture, some of the participants initially had a perception that this was just another ineffective treatment as described by Rugg et al as well [22]. On the contrary, they received proof of the effect as treatment started to affect their bodies. Even small changes in their symptoms were enough to provide proof that acupuncture could actually work.

### Feeling normal

Participants in the present study expressed feelings of being normal. Previous thoughts that PCOS was something untreatable were altered to thoughts of their bodies as capable of normal functions. This is in concordance with another study of acupuncture for patients with rheumatoid arthritis, where they experienced that acupuncture treatment allowed them to feel normal again and regain their lives [24].

## Conclusion

Since acupuncture is a promising treatment for the symptoms of the common syndrome PCOS, the present study adds to the knowledge base by providing the important experiences of patients receiving the treatment. Acupuncture provides a possibility for patients to gain hope as the treatment shows results. The results show that acupuncture empowers the patients to take responsibility for their future well-being, although they may have been initially sceptical to the treatment. As the syndrome had affected them for some time, even small changes offered a chance for them to feel that their bodies were capable of normal function.

## Competing interests

The authors declare that they have no competing interests.

## Authors' contributions

AB performed the interviews, transcribed data, participated in analysis of data and draft of the manuscript. ES-V conceived the idea of the study, participated in analysis of data and draft of the manuscript. Both authors read and approved the final version of the manuscript.

## Pre-publication history

The pre-publication history for this paper can be accessed here:

http://www.biomedcentral.com/1472-6882/12/32/prepub
